# Validation of an LC-MS/MS Method for the Simultaneous Intracellular Quantification of the CDK4/6 Inhibitor Abemaciclib and the EZH2 Inhibitors GSK126 and Tazemetostat

**DOI:** 10.3390/pharmaceutics17040433

**Published:** 2025-03-28

**Authors:** Stefan Senekowitsch, Thomas Freitag, Daniel Dubinski, Thomas M. Freiman, Claudia Maletzki, Burkhard Hinz

**Affiliations:** 1 Institute of Pharmacology and Toxicology, Rostock University Medical Center, Schillingallee 70, 18057 Rostock, Germany; stefan.senekowitsch@med.uni-rostock.de; 2 Department of Medicine, Clinic III—Hematology, Oncology, Palliative Medicine, Rostock University Medical Center, 18057 Rostock, Germany; 3 Department of Neurosurgery, Rostock University Medical Center, 18057 Rostock, Germany

**Keywords:** LC-MS/MS, CDK4/6 inhibitor, EZH2 inhibitor, intracellular accumulation, glioblastoma cells

## Abstract

**Background:** Inhibitors of cyclin-dependent kinases (CDKs) and epigenetic modifier enhancer of zeste homolog 2 (EZH2) have emerged as promising options in the pharmacotherapy of malignant tumors. Recently, we demonstrated synergistic antitumor effects of the CDK4/6 inhibitor abemaciclib and the EZH2 inhibitors GSK126 or tazemetostat in patient-derived glioblastoma (GBM) models. Importantly, all three drugs are substrates of the two most important plasma membrane multidrug transporters ABCB1 and ABCG2, with abemaciclib and tazemetostat also being inhibitors of these proteins. **Methods:** To investigate whether increased intracellular accumulation of either of the two drugs used in combination could have contributed to corresponding synergisms, we developed a simple LC-MS/MS method for simultaneous detection of the three substances in cell culture lysates. The method was validated in accordance with the current International Council for Harmonization of Technical Requirements for Pharmaceuticals for Human Use (ICH) guideline M10 on bioanalytical method validation and study sample analysis. **Results:** All acceptance criteria were met. Subsequent analysis of intracellular drug concentrations confirmed increased cellular uptake of tazemetostat in the presence of abemaciclib in both GBM cell lines studied compared to single agent treatment. A comparable pattern was also observed for GSK126, but in only one of the two cell lines used. **Conclusions:** In conclusion, the observed synergistic antitumor effect could be partly due to increased intracellular accumulation, although this alone is certainly not sufficient to explain it. Overall, the developed method provides a valuable approach for characterizing interactions at the transport level and for predicting the efficiency of both anticancer substance classes in different cell lines.

## 1. Introduction

Cancer therapy has been revolutionized by the introduction of targeted therapies that are more specific and have fewer toxic effects than conventional chemotherapeutics. In addition to monoclonal antibodies, cancer vaccines and gene therapies, small molecules are an important group of modern cancer therapeutics that are part of current research. The mechanisms of action of these small molecules include the inhibition of tyrosine kinases, poly ADP-ribose polymerase (PARP), proteasome or cyclin-dependent kinases (CDK) [1,2,3].

**Figure 1:**
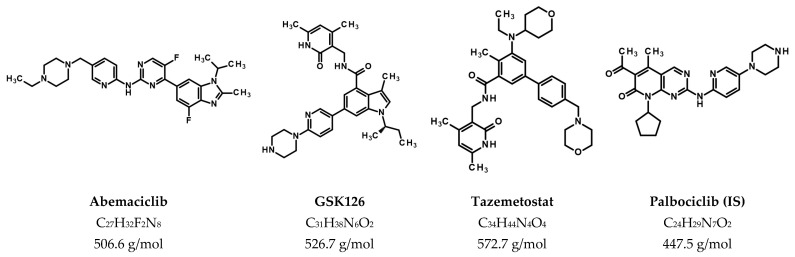
Chemical structure, molecular formulae and average molecular weights of abemaciclib, GSK126, tazemetostat and the internal standard palbociclib.

CDK4 and CDK6 are such targets for cancer therapy. These kinases are responsible for the phosphorylation of the cell cycle repressor protein retinoblastoma (Rb), which leads to the dissociation of E2F transcription factors, thereby promoting the expression of cyclin E. Subsequently, the cells progress from the G1 phase to the S phase, resulting in increased proliferation [4,5]. In several cancer types, this pathway is dysregulated, leading to tumor growth [6,7,8,9]. Abemaciclib (Figure 1), a CDK4/6 inhibitor, has recently been approved for the treatment of hormone receptor-positive, human epidermal growth factor receptor 2-negative advanced breast cancer by the U.S. Food and Drug Administration (FDA) and the European Medicines Agency (EMA) [10,11]. However, abemaciclib is also being investigated in preclinical and clinical studies for the treatment of other tumor types, such as glioblastoma (GBM) [12,13,14,15].

Another relatively new approach to cancer treatment is the inhibition of the epigenetic modifier enhancer of zeste homolog 2 (EZH2) [16]. EZH2 is a histone methyltransferase subunit of the Polycomb 2 repressor complex and serves as a transcriptional regulator. Overexpression or (gain-of-function) mutations of EZH2 have been detected in several cancers, indicating the oncogenic role of EZH2 [17]. In 2020, the FDA approved the EZH2 inhibitor tazemetostat (Figure 1) as a first-in-class drug for the treatment of advanced epithelioid sarcoma or relapsed/refractory EZH2-mutated follicular lymphoma [18]. In addition, other EZH2 inhibitors, such as GSK126 (Figure 1), are being investigated for their antitumor effects [16]. EZH2 is overexpressed in many cases of GBM and contributes to proliferation, migration and invasion [19], so that EZH2 inhibition has been demonstrated to successfully combat GBM cells in several preclinical studies [20,21,22,23].

Recently, we have shown that combined treatment with the CDK4/6 inhibitor abemaciclib and the EZH2 inhibitors GSK126 or tazemetostat led to synergistic antitumor effects in preclinical GBM models [24]. This synergism was attributed to a perturbation of endoplasmic reticulum–mitochondrial homeostasis. However, since abemaciclib [25], as well as GSK126 and tazemetostat [26], are substrates of the ATP-binding cassette (ABC) transporters ABCB1 (P-glycoprotein) and ABCG2 (breast cancer resistance protein, BCRP), with abemaciclib [27] and tazemetostat [28] also being inhibitors of these proteins, these results could also be due to altered cellular uptake of one of the studied drugs when used in combination. Accordingly, a previous study by Wu et al. demonstrated an abemaciclib-induced increase in intracellular accumulation of doxorubicin and rhodamine 123, both substrates of ABCB1 and ABCG2, in transporter-overexpressing cancer cells [27]. Similarly, Sorf et al. observed an accumulation of daunorubicin in HL-60 ABCB1 cells and of mitoxantrone in HL-60 ABCG2 cells after cotreatment with abemaciclib [29]. As a functional consequence of this abemaciclib-mediated interaction, the latter study also showed increased apoptosis of ABCB1-overexpressing CD34-positive peripheral blood mononuclear cells due to the augmented intracellular accumulation of mitoxantrone. The authors of both publications concluded that a combined treatment of ABCB1 and ABCG2 substrates with abemaciclib could have a beneficial antitumor effect in multidrug-resistant cancers. Similar considerations regarding a potential use for combating multidrug-resistant cancer were made for tazemetostat, a triple inhibitor of ABCB1, ABCC1 (multidrug resistance-associated protein 1, MRP1) and ABCG2 [28].

To detect the potential interaction of abemaciclib with GSK126 or tazemetostat at the cellular transport level, a sensitive measurement of intracellular drug concentrations is required. There are a number of published validated liquid chromatography coupled to tandem mass spectrometry (LC-MS/MS) methods for quantifying abemaciclib in human plasma [30], with abemaciclib also detected, along with its metabolites [31,32,33,34,35,36] and/or other breast cancer drugs, such as palbociclib and/or letrozole [33,34,35,36,37,38,39,40,41,42,43]. Furthermore, three validated methods for the quantification of tazemetostat in human [44,45] or rat plasma [46] were described, whereas to the best of our knowledge, no validated methods for the quantification of GSK126 or for the simultaneous analysis of abemaciclib and EZH2 inhibitors have yet been published.

Here, we present a simple LC-MS/MS method for the simultaneous determination of abemaciclib, GSK126 and tazemetostat in cell lysates. The developed method was validated according to the current International Council for Harmonization of Technical Requirements for Pharmaceuticals for Human Use (ICH) guideline M10 on bioanalytical method validation and study sample analysis [47]. Subsequently, the validated method was applied to quantify abemaciclib and the two EZH2 inhibitors in GBM cells treated with the individual substances or their combinations.

## 2. Materials and Methods

### 2.1. Chemicals and Reagents

Abemaciclib and GSK126 were ordered from LKT Laboratories Inc. (St. Paul, MN, USA). Palbociclib was obtained from Chemodex Ltd. (St. Gallen, Switzerland). Tazemetostat was obtained from ApexBio Technology (Houston, TX, USA). LC-MS grade water and LC-MS grade methanol were purchased from Fisher Scientific (Loughborough, UK) and LC-MS grade acetonitrile from scienTEST-bioKEMIX GmbH (Leese, Germany). Formic acid for LC-MS analysis was ordered from Honeywell Fluka (Seelze, Germany). DMSO and Tris-HCl were ordered from Applichem GmbH (Darmstadt, Germany), while HCl (37%) was purchased from Merck KGaA (Darmstadt, Germany).

### 2.2. Stock and Working Solutions

Abemaciclib and GSK126 were prepared as 5 mM stock solutions in dimethyl sulfoxide (DMSO) and tazemetostat was prepared as a 10 mM stock solution, also in DMSO. The palbociclib stock solution was prepared as 1 mM in DMSO, comprising 0.1% 1 M HCl. Stock solutions were stored in aliquots at −80 °C.

Further dilutions were made with methanol. For the internal standard (IS) solution, the palbociclib stock solution was diluted to a concentration of 10 µM. Eight calibration working dilutions were prepared at the following concentrations: 0.10, 0.25, 1.25, 2.50, 5.0, 10.0, 20.0 and 25.0 µM for abemaciclib; and 0.50, 1.25, 6.25, 12.5, 25.0, 50.0, 100 and 125 µM for GSK126 and tazemetostat. The concentrations of the working solutions for quality control (QC) were: 0.10 µM (lower limit of quantification QC, QC-LLOQ), 0.25 µM (low-concentration QC, QC-L), 10.0 µM (medium-concentration QC, QC-M) and 20.0 µM (high-concentration QC, QC-H) for abemaciclib, and 0.50 µM (QC-LLOQ), 1.25 µM (QC-L), 50.0 µM (QC-M) and 100 µM (QC-H) for GSK126 and tazemetostat, respectively. These working solutions were stored at −20 °C and used for no longer than one week.

### 2.3. Sample Preparation

An amount of 200 µL of the thawed sample was transferred into a 1.5 mL microtube (Eppendorf SE, Hamburg, Germany), and 20 µL blank methanol and 20 µL IS working solution (10 µM palbociclib in methanol) were added. For calibration and QC standards, 20 µL of the respective working solutions was added instead of blank methanol to a suspension of lysed A549 cells in 20 mM Tris buffer, pH 7.4. After mixing with a vortexer (Heidolph Reax I, Heidolph Instruments, Schwabach, Germany), 400 µL of cold (−20 °C) acetonitrile was added, followed by further vortexing. Subsequently, the samples were cooled for 10 min at −20 °C to promote protein precipitation. Following centrifugation (16,000× *g*, 5 min, Eppendorf Centrifuge 5415 C, Eppendorf SE, Hamburg, Germany), 500 µL of the supernatant was transferred into 1.5 mL glass vials (CS-Chromatographie Service GmbH, Langerwehe, Germany). Finally, 1 µL of this extract was injected for LC-MS/MS analysis.

### 2.4. LC-MS/MS Instrumentation

The high-pressure liquid chromatography (HPLC) system (Shimadzu, Kyōto, Japan) consisted of a DGU-20A_5R_ degassing unit, two LC-20AD pump units, a SIL-20AC_HT_ autosampler, a CTO-20AC column oven, a CBM-20A communication bus module and an LCMS-8050 mass detector equipped with an electrospray ionization (ESI) unit. Separation was performed using a Multospher^®^ 120 RP8 column (125 × 2 mm, 5 µm particle size, CS-Chromatographie Service GmbH, Langerwehe, Germany), coupled to a corresponding guard column (20 × 2 mm, 5 µm particle size, CS-Chromatographie Service GmbH, Langerwehe, Germany). For the analysis, a linear binary gradient of water with 0.1% formic acid (A) and acetonitrile with 0.1% formic acid at a flow rate of 0.4 mL/min was employed (Table 1). The total runtime was 7.5 min. The oven temperature was set to 40 °C.

For the first 1.85 min and after 4.5 min, the eluate was directed into a waste bottle. Only in between did the flow reach the mass spectrometer. The mass spectrometer was operated in positive multiple reaction monitoring (MRM) mode. In Table 2, the parameters for the detection of the analytes are listed. Furthermore, the following parameters were applied: nebulizing gas flow 3 L/min, heating gas flow 10 L/min, drying gas flow 3 L/min, interface temperature 300 °C, desolvation temperature 526 °C, desolvation line temperature 250 °C and heat block temperature 400 °C. Data acquisition and analysis were carried out using LabSolutions (Version 5.97 SP1, Shimadzu, Kyōto, Japan).

### 2.5. Validation

The method for the quantification of abemaciclib, GSK126 and tazemetostat was validated in accordance with the current ICH guideline M10 on bioanalytical method validation and study sample analysis [47].

#### 2.5.1. Calibration Curve

Calibration standards were prepared using lysates of A549 cells in 20 mM Tris buffer, pH 7.4. For the calibration curves, the peak area ratios of the analytes and the IS were plotted against the analyte concentration. A linear regression with a weighting factor of 1/x^2^ was employed for all analytes. The calibration range was 10–2500 nM for abemaciclib and 50–12,500 nM for GSK126 and tazemetostat, respectively. Overall, eight calibration standards were used for each calibration curve.

The back-calculated concentration for the calibration standards had to be within 85 to 115%, except for the LLOQ (80–120%). Calibration standards that did not fulfill these requirements were excluded from the regression. The calibration curves were accepted if at least six calibration standards met the acceptance criteria and the coefficient of determination (R^2^) was ≥ 0.99.

#### 2.5.2. Accuracy and Precision

In Table 3, the calibration range and the nominal concentrations of the QC levels are shown. QC standards were prepared using lysates of A549 cells in 20 mM Tris buffer pH 7.4, unless stated otherwise. Within-run accuracy and precision were evaluated by analyzing six replicates of each QC concentration level in a single analytical run. Between-run and precision were investigated in three analytical runs over two days (18 samples per QC level). Accuracy had to lie within 85–115%, except for the LLOQ (80–120%). The precision, expressed as the % coefficient of variation (CV), was not allowed to be greater than 15% CV, except for the LLOQ (maximal 20% CV).

#### 2.5.3. Selectivity

For selectivity, six different cell lines (A549, H460, RD, RH30, GBM06 and GBM15) were investigated. The cells were maintained as described below (see Section 2.6). Cell pellets were resuspended in 20 mM Tris buffer, pH 7.4, to obtain a cell concentration of 10^5^ per mL. An amount of 200 µL of each cell suspension was spiked with 20 µL blank methanol twice and further processed as described above (see Section 2.3). For the analytes (abemaciclib, GSK126 and tazemetostat), responses of the blank samples should be below 20% of the response at the LLOQ and for the IS (palbociclib) below 5%, respectively.

#### 2.5.4. Carryover

Carryover was investigated by the injection of blank samples after the calibration standard at the upper limit of quantification (ULOQ). Again, for the analytes (abemaciclib, GSK126 and tazemetostat), responses of the blank samples should be below 20% of the response at the LLOQ and for the IS (palbociclib) below 5%, respectively.

#### 2.5.5. Matrix Effects

Possible matrix effects were investigated by analyzing suspensions of the six aforementioned cell lines at low (QC-L) and high (QC-H) concentrations, each in triplicate. For each cell line, the mean accuracy should be between 85 and 115%. The corresponding precision (% CV) was not allowed to be greater than 15%.

#### 2.5.6. Dilution Integrity

To investigate the dilution integrity, blank matrix was spiked with an appropriate working solution to give a concentration four times the ULOQ. This sample was diluted by a factor of 10 with blank matrix in six replicates. Again, accuracy had to be within the range of 85–115%, while the precision (% CV) should not be greater than 15%.

#### 2.5.7. Stability

The investigations of short-term, long-term and freeze-thaw stability were performed at low and high quality concentration levels (QC-L and QC-H) in four replicates per stability parameter. Freeze–thaw stability was investigated for one, two and three cycles with freezing intervals of at least 12 h at −20 °C. For the determination of long-term stability, samples were stored at −20 °C for two months. The short-term stability of matrix samples was investigated for 2 h at room temperature. Autosampler stability was analyzed by injecting processed matrix samples after storage in the autosampler for 48 h in a run with freshly prepared calibration standards with six replicates at low and high quality concentration levels (QC-L and QC-H).

The acceptance criteria for all stability investigations were met if the calculated accuracy was between 85–115% and the precision (% CV) was not greater than 15%.

#### 2.5.8. Reinjection Reproducibility

The reinjection reproducibility was investigated by reinjecting a run of calibration and QC samples. The determined concentration values from the second run were compared to those from the initial run. Again, the acceptance criteria were 85–115% (for LLOQ 80–120%) for accuracy and a maximal 15% CV (for LLOQ 20%) for precision.

#### 2.5.9. Recovery Rate

The recovery rates for the analytes and the IS were analyzed at low, medium and high quality control concentration levels (QC-L, QC-M and QC-H) with four replicates. For this purpose, the areas of the post-extraction spiked samples were compared with the areas of the pre-extraction spiked samples. The results were acceptable if the % CV of the determined recovery rates was not greater than 15%.

### 2.6. Cell Culture

A549 and RH30 cells were purchased from Deutsche Sammlung von Mikroorganismen und Zellkulturen GmbH (DSMZ, Braunschweig, Germany; A549: DSMZ no. ACC 107; RH30: DSMZ no. ACC 489). H460 and RD cells were obtained from the American Type Culture Collection (ATCC; H460: ATCC no. HTB-177; RD: ATCC no. CCL-136). These four cell lines were used only for analytical purposes (validation of selectivity and matrix effects, A549: calibration, QC and validation). The maintenance procedures of the different cell lines can be found in previous publications [48,49]. The GBM cell lines GBM06 and GBM15 were established from GBM tumor samples obtained from patients at the Department of Neurosurgery of Rostock University Medical Center, Germany, as described previously [24]. For cell culture, Dulbecco’s Modified Eagle Medium (DMEM)/Ham’s F12 medium was supplemented with 10% fetal calf serum, 6 mM l-glutamine and 1% penicillin/streptomycin (all from PAN-Biotech, Aidenbach, Germany) and incubated at 37 °C in a humidified atmosphere of 5% CO_2_.

GBM cells (1 × 10^5^ cells/well) were seeded in six-well plates and incubated overnight. The DMSO stock solutions of the test substances were diluted with Dulbecco’s phosphate-buffered saline (DPBS). The final solvent concentration per compound in the cell culture incubates was 0.1% (*v*/*v*) DMSO. An amount of 5 mL of medium, containing either 1 µM abemaciclib, 10 µM GSK126, 10 µM tazemetostat or a combination of 1 µM abemaciclib plus 10 µM GSK126 or 10 µM tazemetostat, was added and incubated for 72 h. Following incubation, the medium was removed and the cells were washed with DPBS. After removal of the DPBS, the six-well plates were frozen at −80 °C.

For the analysis of intracellular concentrations of abemaciclib, GSK126 and tazemetostat, the six-well plates were brought to room temperature. Per well, 1 mL of 20 mM Tris buffer with a pH of 7.4 was added. The cells were scraped with a natural rubber wiper (Carl Roth GmbH + Co., KG, Karlsruhe, Germany) and the cell suspension was transferred into a 1.5 mL microtube. For cell lysis, the reaction tubes were frozen at −20 °C. Following thawing, the samples were processed as described above (see Section 2.3). The protein content of the samples was determined using a bicinchoninic acid (BCA) protein assay kit (Thermo Fisher Scientific Inc., Rockford, IL, USA).

## 3. Results

### 3.1. Validation of the LC-MS/MS Method

**Figure 2:**
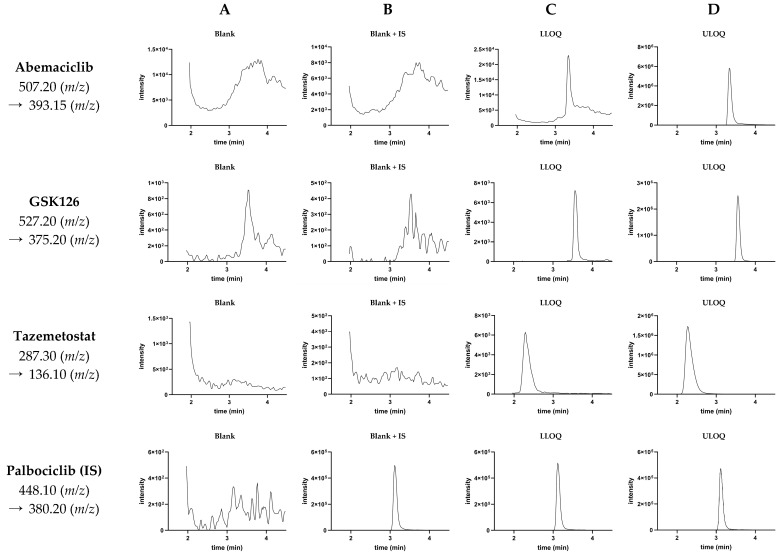
Representative chromatograms of cell suspensions in 20 mM Tris buffer, pH 7.4, spiked with blank methanol (**A**), only palbociclib (IS) (**B**) or palbociclib (IS) and the three analytes (abemaciclib, GSK126, tazemetostat) at lower limit of quantification (LLOQ, (**C**)) or upper limit of quantification (ULOQ, (**D**)). The mass transition that applies to the respective line of chromatograms is listed on the left.

The developed method for the quantification of abemaciclib, GSK126 and tazemetostat had a runtime of 7.5 min. Representative chromatograms of blank samples (double-blank and blank plus IS) and calibration standards at the LLOQ and ULOQ are shown in Figure 2.

The method was validated for the determination of abemaciclib, GSK126 and tazemetostat in cell lysates in 20 mM Tris-HCl buffer at a pH of 7.4. There were no interfering peaks in the blank samples of the six different cell lines examined, indicating that the method developed was selective for the analytes. The injection of a blank sample following an analytical run of the highest calibration standard also did not result in any interfering peaks for any of the analytes. Carryover did not pose a problem.

**Figure 3:**
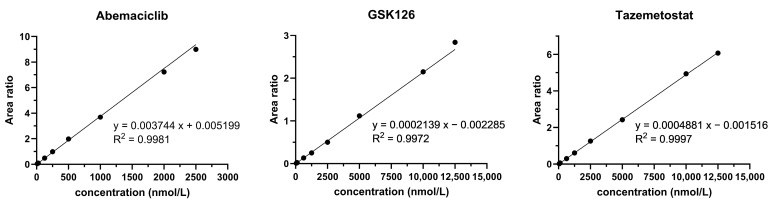
Representative calibration curves of abemaciclib, GSK126 and tazemetostat.

All calibration curves were weighted by 1/x^2^ and linear with R^2^ > 0.99. At least six calibration standards per run met the acceptance criteria regarding the back-calculated concentration. In Figure 3, exemplary calibration curves are presented. As shown in Table 4, the acceptance criteria for within-run and between-run accuracy (85–115%, 80–120% at LLOQ) and precision (% CV < 15, % CV < 20 at LLOQ) were also fulfilled.

The recovery of the analytes and the IS was high (mean recovery ≥ 94.9%) and reproducible (% CV ≤ 9.16%) at all concentrations examined (QC-L, QC-M and QC-H). The mean accuracy of the matrix effect studies ranged from 88.8 to 102.1% for abemaciclib, from 85.3 to 105.9% for GSK126 and from 90.7 to 103.7% for tazemetostat, while the corresponding precision values (% CV) were not greater than 13.27%, 11.02% and 11.14%, respectively. The accuracy and precision of samples spiked at a concentration of 4× ULOQ and diluted 10-fold with blank matrix were 110.2% and 4.01% for abemaciclib, 102.3% and 5.94% for GSK126, and 110.3% and 5.62% for tazemetostat. The reinjection of an analytical run was reproducible with mean accuracies ranging from 88.2% to 101.1% for abemaciclib, 92.4% to 98.9% for GSK126 and 98.0% to 104.2% for tazemetostat, with the corresponding precision values (% CV) not greater than 6.09%, 5.51% and 2.85%, respectively.

The results of the stability investigation are summarized in Table 5. Abemaciclib, GSK126 and tazemetostat were stable under all conditions tested.

### 3.2. Intracellular Concentrations of Abemaciclib, GSK126 and Tazemetostat After Single and Combined Treatment of Patient-Derived GBM Cells

**Figure 4:**
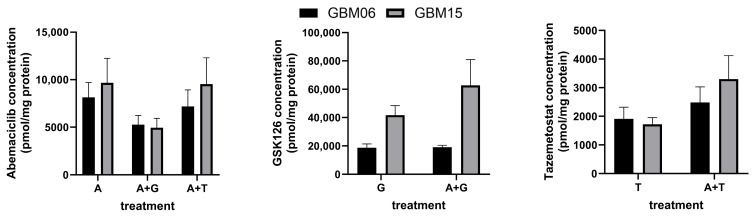
Intracellular concentrations of abemaciclib (A), GSK126 (G) and tazemetostat (T) in GBM06 and GBM15 cells. Cells were incubated with either 1 µM abemaciclib, 10 µM GSK126, 10 µM tazemetostat or a combination of 1 µM abemaciclib plus 10 µM GSK126 or 10 µM tazemetostat for 72 h, followed by lysis and LC-MS/MS analysis of intracellular drug concentrations. Data are mean + SD of *n* = 4 independent experiments.

Finally, the validated LC-MS/MS method was applied to GBM06 and GBM15 cells that were treated with abemaciclib, GSK126 or tazemetostat. The results are shown in Figure 4.

Abemaciclib concentrations were lower after the combined treatment with GSK126 in both cell lines, while there was virtually no change following the combined treatment with tazemetostat. In the GBM06 cells, GSK126 concentrations were unaffected by the combination with abemaciclib. In contrast, there was a rise in the intracellular GSK126 concentration in the GBM15 cells after the combined treatment. Compared to GSK126, tazemetostat accumulated to a much lesser extent in the GBM cells. The combination with abemaciclib led to an increase in intracellular tazemetostat concentrations in both GBM cell lines.

## 4. Discussion

In the present work, we established and validated an LC-MS/MS method for the quantification of abemaciclib, GSK126 and tazemetostat in cell lysates in 20 mM Tris-HCl pH 7.4. The developed method is characterized by simple sample preparation through protein precipitation and a short runtime of 7.5 min. The eluent was directed to a waste bottle for the first 1.85 min and after 4.5 min to reduce the contamination of the ionization unit and the risk of possible matrix effects. The applied gradient reached a high proportion of 95% of the organic phase (acetonitrile with 0.1% formic acid) to reduce the intense peak tailing of abemaciclib and to clean the column from residues from the analytes as well. Compared to a RP18 column (Multospher^®^ 120 RP18 AQ column, 250 × 2 mm, 5 µm particle size, CS-Chromatographie Service GmbH, Langerwehe, Germany), which was also used in preliminary testing, the applied RP8 column provided improved peak shapes while ensuring sufficient retention of abemaciclib, GSK126 and tazemetostat. During method development, sample preparation by liquid–liquid extraction with ethyl acetate and n-hexane was also tested. However, as the detector signals were sufficient and there was no interfering matrix effect after protein precipitation with acetonitrile, this procedure was retained.

The mass-spectrometric parameters were optimized by injecting standard solutions of the analytes in methanol into the LCMS system without a column (flow injection analysis). The optimization was conducted by applying the built-in program for method optimization of the LabSolutions software (Version 5.97 SP1, Shimadzu, Kyoto, Japan) used. The mass transition used for abemaciclib quantification (507.20 (*m/z*) → 393.15 (*m/z*)) showed a relatively intense noise rate, as shown in Figure 2. However, the signal intensities of abemaciclib were very high, so the LLOQ of 10 nM for abemaciclib did not pose a problem. For abemaciclib, extensive carryover effects have been reported [30,32,37,40]. The gradient used in the current method reached a proportion of 95% of the organic mobile phase (acetonitrile with 0.1% formic acid). Under these conditions, we did not observe relevant carryover effects. Interestingly, for tazemetostat (molar mass of 572.7 g/mol), higher signal intensities were monitored for the double-charged positive parent ion in the ESI. Thus, this parent ion mass/charge ratio (*m/z* 287.30) was used for further method development and validation. Palbociclib was chosen as the internal standard for quantification as it provides similar physicochemical properties to the analytes in regard to its molecular weight and its alkaline acid–base behavior. The application of isotopically labeled internal standards could have possibly further enhanced the reliability of the method. However, the developed method fulfilled all the acceptance criteria of the validation process.

The subsequent application of the developed and validated method for the intracellular determination of the three analytes in correspondingly treated GBM cells revealed a number of remarkable findings. First, although the same concentrations were used to incubate the cells, the resulting intracellular concentrations of tazemetostat were much lower than those of GSK126. Taking into account the similar potencies of GSK126 and tazemetostat for EZH2 inhibition [17,50], this fact could explain the lower toxicity of tazemetostat compared to GSK126 in GBM cell lines, as observed in the aforementioned study investigating the effects of combining abemaciclib with EZH2 inhibitors [24]. However, it has to be considered that abemaciclib, GSK126 and tazemetostat may have been transformed to metabolites that were not quantified by the applied LC-MS/MS method. Second, despite tazemetostat being an inhibitor of ABCB1, ABCC1 and ABCG2 [28] and abemaciclib being a substrate of ABCB1 and ABCG2 [25], the intracellular abemaciclib concentrations were not affected by the combined treatment. Possibly, intracellular tazemetostat concentrations were not sufficient to inhibit ABC transporters, or the inhibitory effects of tazemetostat were equalized by a concurrent EZH2-independent (off-target) upregulation of ABCG2 expression by the EZH2 inhibitor, as reported in another study [51]. The lower intracellular abemaciclib concentrations following combined treatment with GSK126 would support the latter assumption, since the same investigation also demonstrated an increase in ABCG2 expression by GSK126 [51]. Third, the simultaneous treatment with abemaciclib seems to have caused an increase in the intracellular accumulation of both EZH2 inhibitors. As explained before, this could be a result of the inhibition of ABCB1 and ABCG2 by abemaciclib [27,29], as both EZH2 inhibitors are substrates of ABCB1 and ABCG2 [26]. However, the extent to which this increase in intracellular concentrations of the EZH2 inhibitors contributed to the synergistic antitumor effectivity in GBM cell lines remains unclear. The changes in intracellular accumulation of the EZH2 inhibitors following combined treatment with abemaciclib were different for the two investigated GBM cell lines, possibly related to differences in the expression of the ABC transporters. While the combination of GSK126 with abemaciclib led to an increase in intracellular GSK126 concentrations of 30% only in GBM15 cells, the reported highly synergistic effects were present in GBM06 and GBM15 cells. The intracellular accumulation of tazemetostat increased by 51% and 92% in GBM06 and GBM15 cells, respectively, following combined treatment with abemaciclib. At the same time, the synergistic effects were not as distinct compared to the combination of GSK126 and abemaciclib [24]. Overall, the synergistic antitumor effects of combining abemaciclib with EZH2 inhibitors appear to be due, in part but not entirely, to increased accumulation in GBM cells.

In summary, we have developed and validated a simple and reliable method for the simultaneous intracellular detection of the CDK4/6 inhibitor abemaciclib and the EZH2 inhibitors GSK126 and tazemetostat. This is an essential prerequisite for further investigation of both drug classes with regard to possible interactions at the transport level in a variety of tumor entities.

## Figures and Tables

**Table 1:** Linear binary gradient for the analysis of abemaciclib, GSK126 and tazemetostat (eluent A: water with 0.1% formic acid; eluent B: acetonitrile with 0.1% formic acid).

Time (min)	Eluent B (%)
0.00	20
2.00	95
4.00	95
4.10	20

**Table 2:** Mass spectrometric parameters for the detection of tazemetostat, abemaciclib, GSK126 and palbociclib.

Analyte	Precursor Ion (*m/z*)	Product Ion (*m/z*)	Collision Energy (eV)	Retention Time (min)
Abemaciclib	507.20	393.15	−24.0	3.30
GSK126	527.20	375.20	−27.0	3.60
Tazemetostat	287.30	136.10	−15.0	2.30
Palbociclib (IS)	448.10	380.20	−29.0	3.10

**Table 3:** Calibration range and quality control level concentrations of abemaciclib, GSK126 and tazemetostat.

Analyte	Calibration Range (nmol/L)		Quality Control Level Concentration (nmol/L)			
	LLOQ	ULOQ	QC-LLOQ	QC-L	QC-M	QC-H
Abemaciclib	10	2500	10	25	1000	2000
GSK126	50	12,500	50	125	5000	10,000
Tazemetostat	50	12,500	50	125	5000	10,000

LLOQ, lower limit of quantification; ULOQ, upper limit of quantification; QC, quality control; L, low concentration; M, medium concentration; H, high concentration.

**Table 4:** Within-run and between-run accuracy and precision.

Parameter	Quality Control Level	Abemaciclib	GSK126	Tazemetostat
Within-run accuracy (%, mean of *n* = 6)	QC-LLOQ	106.4	110.9	100.7
	QC-L	93.3	89.8	88.5
	QC-M	93.4	94.2	92.8
	QC-H	93.3	98.8	94.9
Within-run precision (% CV of *n* = 6)	QC-LLOQ	3.33	3.21	4.53
	QC-L	4.16	9.35	6.32
	QC-M	1.74	1.35	1.57
	QC-H	4.34	4.10	4.02
Between-run accuracy (%, mean of *n* = 18)	QC-LLOQ	107.2	110.7	103.3
	QC-L	92.8	90.0	92.2
	QC-M	89.1	90.5	90.2
	QC-H	101.3	103.3	97.4
Between-run precision (% CV of *n* = 18)	QC-LLOQ	4.65	4.00	4.12
	QC-L	4.20	6.23	5.14
	QC-M	4.94	4.29	3.26
	QC-H	6.55	4.63	3.80

CV, coefficient of variation; LLOQ, lower limit of quantification; QC, quality control; L, low concentration; M, medium concentration; H, high concentration.

**Table 5:** Stability of abemaciclib, GSK126 and tazemetostat.

Storage Condition	Quality Control Level	Abemaciclib		GSK126		Tazemetostat	
		Accuracy (%)	Precision (% CV)	Accuracy (%)	Precision (% CV)	Accuracy (%)	Precision (% CV)
Short-term stability (room temperature, 2 h)	QC-L	108.0	3.38	101.4	3.73	102.1	5.22
	QC-H	99.9	3.56	104.9	4.51	101.5	4.09
Long-term stability (−20 °C, 2 months)	QC-L	103.9	1.77	90.28	7.21	99.5	2.41
	QC-H	100.6	2.17	87.0	1.21	100.3	1.08
Freeze–thaw-stability (3 cycles, −20 °C)	QC-L	102.4	4.79	90.8	5.21	95.9	3.33
	QC-H	94.3	1.53	92.7	4.57	93.6	1.71
Autosampler stability (10 °C, 48 h)	QC-L	90.4	7.57	87.2	5.53	86.5	5.39
	QC-H	86.6	4.69	93.6	7.07	87.2	4.89

CV, coefficient of variation; QC, quality control; L, low concentration; H, high concentration). Data were obtained from *n* = 4 (short-term, long-term, freeze–thaw stability) or *n* = 6 (autosampler stability) determinations.

## Data Availability

Data are available from the first author upon reasonable request.
